# Emotion Recognition in Adolescents with Down Syndrome: A Nonverbal Approach

**DOI:** 10.3390/brainsci7060055

**Published:** 2017-05-23

**Authors:** Régis Pochon, Claire Touchet, Laure Ibernon

**Affiliations:** 1 Cognition, Health and Socialization Laboratory, Higher School of Teaching and Education, University of Reims Champagne-Ardenne, 51097 Reims, France; 2 Research Center in Psychology: Cognition, Psyche and Organizations, University of Picardie Jules Verne, 80025 Amiens, France; claire.touchet@u-picardie.fr (C.T.); laure.ibernon@u-picardie.fr (L.I.)

**Keywords:** down syndrome, emotion recognition, non-verbal recognition

## Abstract

Several studies have reported that persons with Down syndrome (DS) have difficulties recognizing emotions; however, there is insufficient research to prove that a deficit of emotional knowledge exists in DS. The aim of this study was to evaluate the recognition of emotional facial expressions without making use of emotional vocabulary, given the language problems known to be associated with this syndrome. The ability to recognize six emotions was assessed in 24 adolescents with DS. Their performance was compared to that of 24 typically developing children with the same nonverbal-developmental age, as assessed by Raven’s Progressive Matrices. Analysis of the results revealed no global difference; only marginal differences in the recognition of different emotions appeared. Study of the developmental trajectories revealed a developmental difference: the nonverbal reasoning level assessed by Raven’s matrices did not predict success on the experimental tasks in the DS group, contrary to the typically developing group. These results do not corroborate the hypothesis that there is an emotional knowledge deficit in DS and emphasize the importance of using dynamic, strictly nonverbal tasks in populations with language disorders.

## 1. Introduction

Knowing how to correctly identify the emotion expressed by someone else’s face is an essential capacity for the proper development of interpersonal relationships during childhood [1,2]. This capacity is an emotional competency; such competencies allow for smooth adjustment in social interactions. In addition, emotion-understanding deficits can create social adaptation problems, complicate integration into the school environment, and may ultimately compromise academic success [3]. This developmental domain has been examined by many studies of people with autism spectrum disorder (ASD) and to a lesser degree in people with intellectual disabilities (ID). Today, development in ID is mainly studied with an etiology-based approach in order to accurately describe the pattern of behavioral consequences associated with a given disorder or syndrome throughout development [4,5]. The main idea underlying this approach is that a better understanding of pathologies, particularly differences in developmental trajectories, makes it possible to better target preventive, educational or therapeutic interventions.

In the case of Down syndrome (DS), the study of socio-emotional competencies has only recently aroused much research interest, compared to the study of cognitive and linguistic characteristics. Undoubtedly, one explanation is the positive stereotype that describes people with DS as affectionate and highly sociable [6]. The strong interest children with DS display for other people’s faces has also been highlighted [7,8], which led Kasari et al. [9] to hypothesize that these children have an advantage in emotional facial expression recognition compared to other children with ID. Their actual results contradicted this hypothesis, as they revealed deficits in children with DS who had a mean developmental age (DA) of 3.35 years and a mean chronological age (CA) of 6.39 years. Those results corroborated the slightly earlier conclusions of Wishart and Pitcairn [10] regarding older children (unspecified mean DA and mean CA of approximately 12 years). These findings led to further studies on emotion recognition in DS and in other areas of socio-emotional competency such as empathy [11] and emotional regulation [12]. Regarding the recognition of emotional facial expressions, the summary by Cebula et al. [13] reported on the problems experienced by children and adults with DS in different studies, which could be related to specific features noted in studies of social referencing [14,15]. In children and adolescents with DS, the deficits reported in various studies relate essentially to the recognition of fear, surprise and anger [10,16,17,18]. Cebula et al. [13] emphasize that, unlike what has been found in ASD, these are subtle differences that only appear in comparison with typically developing (TD) children of the same DA and not when the comparison is with other participants with IDs of different etiologies. These authors also point out that this emotion recognition problem is one of many subtle differences that have been found in children with DS in a variety of areas of social cognition.

In adults with DS, previous findings revealed conflicting results: Hippolyte et al. [19,20] found problems in recognizing surprise, sadness and also neutral expression while Carvajal et al. [21] reported no specific difficulties processing emotional expressions. As Cebula et al. [13] pointed out, there is still a lack of experimental evidence to conclude that there is a specific emotion recognition profile characterizing DS. The aim of our study is to contribute to a better understanding of the processes involved in the recognition of emotional facial expressions by adolescents with DS.

Moore’s [22] recommendations for the study of emotion recognition in persons with ID led to efforts to minimize the cognitive and verbal load in assessing children with DS [17,18], but those studies were not completely free of the use of emotional vocabulary since, for example, the experimenter named the emotions that were presented. On the other hand, as Channell et al. [23] pointed out, the tasks used in earlier studies evaluated recognition on the basis of photographs of static facial expressions, which does not allow one to conclude that people with DS have a true deficit affecting the recognition of emotional facial expressions. Tasks that use stimuli that are closer to real-life interaction situations and richer in emotional information are necessary to continue the study of emotion recognition.

Pochon and Declercq [24] presented the results of a longitudinal study in which a task of nonverbal recognition was proposed: children with DS had to recognize basic emotions on the basis of an emotional vocalization rather than an emotional label. With this method, the DS group obtained similar results to a TD group with the same nonverbal DA. The replication of this study with the same participants, the same visual material (photographs) and the same procedure (but soliciting recognition on the basis of emotional labels) revealed deficits in participants with DS in comparison to TD participants with the same DA [25]. The study presented here continues the investigation of the ability to recognize basic emotional facial expressions by means of a nonverbal protocol that uses dynamic stimuli (video clips) rather than static ones (photographs). Dynamic emotional stimuli were used with children and adolescents with DS in two recent studies, which produced contradictory results. In their study, Channell et al. [23] presented three emotions (happiness, sadness and fear) in real filmed sequences. Their results showed no deficit in emotion recognition of children with DS compared with TD children of the same level on the Peabody Picture Vocabulary Test (PPVT-4) [26]. Conversely, in the study by Martínez-Castilla et al. [27], participants with DS obtained worse results on the Animated Full Facial Expression Comprehension Test [28] than TD participants and participants with Williams syndrome (WS) with the same DA (Stanford–Binet test, [29]). The stimuli used were animations created by morphing photographs of expressive faces taken from Ekman and Friesen’s [30] set. It should be noted that this study mobilized a greater linguistic load since participants had to produce a verbal response involving emotional vocabulary, while in the study by Channell et al., [23] participants simply had to respond by pointing to a schematic face from among three faces presented and named earlier by the investigator.

The aim of this study was to continue the investigation of emotional facial expression recognition abilities in adolescents with DS by using dynamic material, namely filmed sequences presenting the faces of professional actors expressing six basic expressions (happiness, sadness, fear, anger, disgust, and surprise). The originality of our method resides in the fact that it relies on a so-called “nonverbal” task in which emotional vocabulary (i.e., the words referring to emotions) is never used to trigger recognition, which is done instead on the basis of the actor’s voice intonation and facial expression. The verbal load induced by responding is therefore nil, since the sentences spoken by the actors, which were identical for all emotions, were either meaningless or had no emotional content. On the other hand, selection of the right answer only involved choosing between two video clips, which avoided overloading working memory. Thus, this task is suitable for people with DS, whose language and short-term memory problems are well known [31,32,33]. It also takes account of Moore’s [22] recommendations regarding the ecological validity of emotional stimuli presented to people with ID.

Two approaches are taken to data analysis: first, the comparison of individually matched groups, then the study of cross-sectional developmental trajectories. We will start by comparing the level of recognition of emotional facial expressions in adolescents with DS and TD children of the same nonverbal level. Given that the protocol contains emotions that people with DS found difficult to recognize in earlier studies (fear, disgust and anger), we hypothesized that the overall level of emotional facial expression recognition would be significantly lower in the DS group than in the TD group of the same nonverbal level. More specifically, given the difficulties with certain emotions reported in the literature, we expected poorer results in adolescents with DS for expressions of fear, surprise and anger. The study of cross-sectional developmental trajectories then made it possible to locate the onset of the trajectories and the progression of emotional facial expression recognition as a function of the level of nonverbal reasoning assessed with Raven’s Colored Progressive Matrices (RCPM) [34].

## 2. Materials and Methods

### 2.1. Participants

Twenty-four adolescents with DS aged from 10.5 to 18.9 years old took part in this study (14 boys, 10 girls). They were being educated in specialized institutions (20) or in the regular school system with accompaniment by a specialized service (4). The diagnosis of trisomy 21 was confirmed by the medical teams at the institutions where these adolescents were being monitored. Initially, 30 participants with DS were recruited, but only the ones who were able to accurately understand the instructions during pretests were included in the study. As well, adolescents with sensory deficits, major attention disorders or ASD were not included. Another group of 24 TD children aged from 3.5 to 5.5 years old were recruited in kindergartens and elementary schools (17 boys, 7 girls). They did not have any sensory, physical or mental developmental disorders and had not experienced any significant educational delay. These 24 TD children were selected from a sample of 69 children aged 3.5 to 10 years (*m* = 6.57; SD = 1.93) who had participated in experimental tasks in a previous study [35], this sample of 69 children will be the reference sample for the analysis of developmental trajectories. The TD group was created by individual matching with participants in the DS group for nonverbal reasoning as measured by the gross score on the RCPM [34]. The characteristics of each group are presented in Table 1. The ability to efficiently process faces, which can impact success on emotional facial recognition tasks, was measured with the Benton Facial Recognition Test (BFRT) [36], and this measure was taken into account in the matching. This is a standardized test that measures the ability to identify unfamiliar faces; the two groups were at similar levels (*t*(45) = −0.83, *p* = 0.41). Finally, to assess their nonverbal DA, the participants with DS completed the Cubes subtest (block design) of the French version of the Wechsler Preschool and Primary Scale of Intelligence (WPPSI-III) [37]. The mean DA of this group was 4.49 years, which is very close to the mean CA of the TD group (4.43 years).

### 2.2. Ethical Approval

Ethical approval was not required for this study in accordance with the national and institutional guidelines. This study was carried out in accordance with the recommendations of French law written informed consent from all subjects. The participants’ parents and the participants themselves gave their consent to participate in this study in accordance with the Declaration of Helsinki. Informed consent was obtained in writing from all the participants with DS after the experimenter read aloud the document presenting the protocol and explained the nature of the tests. The participants’ parents or guardians also gave their consent in writing after being informed of the objectives of the study, the nature of the tests to be administered and the fact that they could withdraw from the study at any time. The administrators at the medical, social, and educational institutions also gave their consent after the research project was presented, since the meetings took place at the educational institutions.

### 2.3. Tasks and Procedure

Two original experimental tasks with similar design were especially created. We used dynamic stimuli and language skills were not required. These tasks, with similar designs, had been the subject of a preliminary study in children aged 3 to 11 years to ensure their developmental sensitivity and collect typical developmental data [35]. Preliminary tests showed that they were well suited to people with intellectual disability. 

#### 2.3.1. Control Task

Six familiar objects were used for this task: a small plastic bottle, a ceramic bowl, a metal cooking pot, a stemmed glass, a plastic citrus juicer, and a plastic kitchen spatula. Each one appeared three times as a target. Presented in short video clips (3.2 s), each object was struck by another object: nine times with a large wooden spoon and nine times with a large metal spoon, so different sounds were produced. The objects were hit in three different ways: three blows, two double-blows or three double-blows. Each method was used six times (Table 2). During each presentation, two video clips were presented simultaneously depicting the same hitting object and the same hitting method; the only difference was the object that was hit (i.e., a target object and a distractor). A single soundtrack, corresponding to the target video clip, was played with a lag (desynchronization) so participants could not use the synchronization of the sound with the object’s movements as a cue. For instance, two video clips (one target and one distractor) were simultaneously displayed: on the left side of the screen a video showed a bowl and on the right side another video showed a small bottle; on each of these two videos, the presented object was being struck twice with a large wooden spoon. While the videos were playing, the participant only heard the soundtrack of the target video with a time lag.

Participants were asked to associate the sound they heard with the corresponding video clip. The video clips were presented side by side with a small space between them, and were played after the presentation of a target to encourage the participant to gaze at the center of the screen. They were played in a loop until the participant responded. To promote task engagement, the experimenter sat next to the participant, was looking at the screen, and if necessary provided verbal encouragement. Responses were given by manual pointing at the screen of a portable computer (Hewlett-Packard, Palo Alto, CA, USA) (15”, resolution of 1366 × 768 pixels). The maximum score on this task was 18 (six target objects presented three times).

#### 2.3.2. Emotional Task

Six basic emotional facial expressions were presented during this task: happiness, sadness, anger, disgust, surprise and fear. Each one was the target emotion three times. At each presentation, the emotions were expressed by the same actor (nine times by a man, nine times by a woman) with only the head and shoulders visible. The two actors were trained to express these emotions as needed by the task and the video clips that were used were selected from a large number of takes: they had to be correctly identified in 95% of cases by 20 non-expert adults aged 20 to 40 years. The actors alternately spoke three sentences in French that either had non-emotional content (“Léa came in a plane”; “The bottle is on the table”) or were made up of nonwords (“Cognogo tiketou”). These sentences, each of which was used six times, were spoken with the appropriate prosody and facial expression for each emotion. At each presentation, the same actor (speaking the same sentence) appeared in both video clips; only the emotion expressed by his/her face and voice differed. For example, the screen simultaneously displayed two video clips of the same actor: on the left side of the screen with an angry face and on the right side with a scared face; on each of these two videos, the actor was pronouncing the same sentence; while the videos were playing, the participant only heard the sentence of the target video with a time lag. To respond correctly, participants had to associate the prosody of the sentence they heard with the corresponding facial expression. The stimuli were presented with a method identical to that used in the control task, including the soundtrack lag, which prevented participants from relying on the movements of the actors’ lips. The maximum score on this task was also 18 (six target emotions presented three times).

Participants were tested in a quiet, familiar room at their care institution, school or home. Administration of all tasks took from 70 to 90 min, and the tasks were divided among three sessions lasting 20 to 30 min each so that the results would not be affected by fatigue, boredom or concentration problems. Task administration was divided into four blocks: one learning block and three experimental blocks. Each block was comprised of 12 items, six from the control task and six from the emotional task, presented alternately. The purpose of the learning block was to teach participants the task and ensure they understood it properly. If, at the end of this block, the participant still could not understand the task, the task administration was interrupted. A pause after each block made it possible to chat with participants and keep them motivated. The initial instructions for the control items were as follows: “Listen carefully to me. Now, when I press this button, you’ll see two short films, one on the left and the other on the right (the examiner shows the locations on the blank screen). At the same time as you’re watching these two little films, you’ll hear a sound. You have to point your finger at the film that goes with the sound we hear—the one on the left or the one on the right. Do you understand? Now, we’re starting—are you ready? Watch this target closely.” For the emotional items, the initial instructions were as follows: “Listen carefully to me. Now, when I press this button, you’ll see two short films, one on the left and the other on the right (the examiner shows the locations on the blank screen). At the same time as you’re watching these two little films, you’ll hear someone talking. You have to point your finger at the film where the person is talking—the one on the left or the one on the right. Do you understand? Now, we’re starting—are you ready? Watch this target closely.” Throughout the task, no emotion words were used. It very soon became unnecessary to repeat the instructions in full.

## 3. Results

The results were analyzed with two approaches: first, the individual matching method in which an analysis of variance (ANOVA) was used to compare the emotion recognition abilities of adolescents with DS and TD children. The second method was the study of the cross-sectional developmental trajectories related to emotion recognition: first for all emotions and then for each emotion separately, with reference to typical data obtained from children aged 3 to 10.

### 3.1. Comparison of Results of Groups Matched for Nonverbal Level (RCPM)

The normality of distributions for each variable studied was tested for each group using one-sample Kolmogorov–Smirnov tests. The distribution was normal for the overall results on the control and emotional tasks, which made it possible to conduct the ANOVA followed by a post hoc test (Tukey’s test). On the other hand, within each experimental task, the distribution of scores for each emotion (emotional task) did not follow a normal distribution, so we conducted non-parametric analyses.

**Figure 1:**
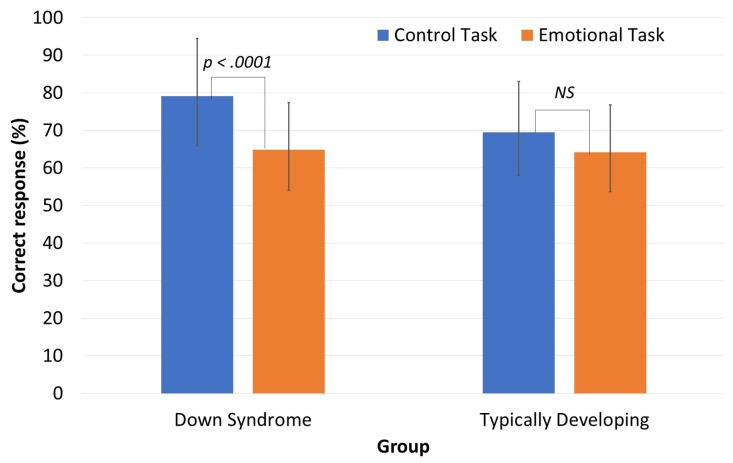
Mean percentage of correct responses in the experimental tasks for the two groups.

The results for the two experimental tasks (Figure 1) were analyzed with a mixed-design ANOVA with Group as the between-subjects variable (DS, TD) and Task as the within-subjects variable (control, emotional). There was no significant main effect of Group ((*F*(1,46) = 2.417, *p* = 0.127, *η_p_^2^* = 0.049)). A significant main effect of Task ((*F*(1,46) = 23.976, *p* = 0.001, *η_p_^2^* = 0.343)) was found but it was qualified by a significant Group x Task interaction ((*F*(1,46) = 5.047, *p* = 0.029, *η_p_^2^* = 0.099)). Post hoc comparisons did not reveal any intergroup differences, regardless of task, but intragroup comparisons showed that the DS group performed significantly better on the control task than the emotional task (*p* < 0.001, *d* = 1.15), whereas this was not the case for the TD group (*p* = 0.331).

Performance per emotion (Table 3) showed that the DS group scored higher than the TD group for the emotions of happiness and sadness while they exhibited very similar results for the others. However, these results are given for descriptive purpose only.

### 3.2. Study of Developmental Trajectories for Emotion Recognition

For these analyses, the entire sample of TD children (*n* = 69) was used. Consequently, the typical developmental trajectories were constructed on the basis of CAs ranging from 3.5 to 10 years (*m* = 6.57; SD = 1.93). This enabled us to cover an essential proportion of the age range during which the recognition of basic emotions is developed [38]. Moreover, this age range covers the nonverbal DAs of participants in the DS group.

**Figure 2:**
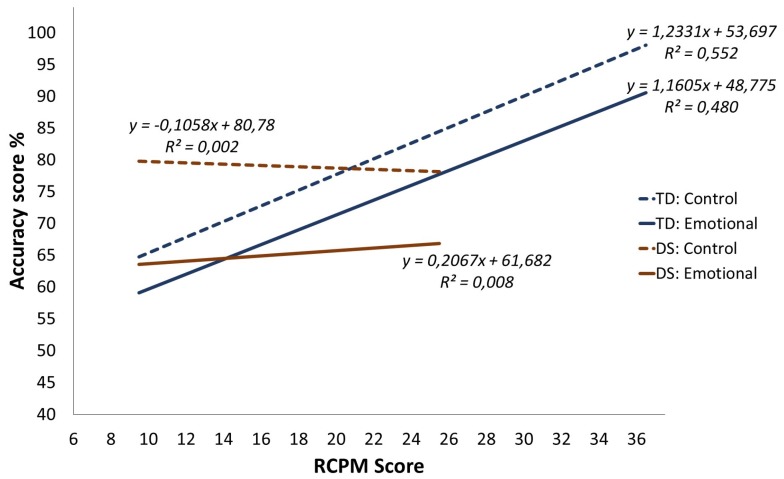
Cross-sectional developmental trajectories for the experimental tasks over Raven’s Colored Progressive Matrices (RCPM) scores.

First, the developmental trajectories for emotion recognition (according to the RCPM) were identified. Figure 2 shows the differences in the two groups’ performance on the two experimental tasks (control and emotional). The aim is to characterize the DS group’s trajectory in comparison to the typical developmental trajectory. As recommended by Thomas et al. [39], the comparisons for each experimental task were done using an analysis of covariance (ANCOVA) with score on the task as the dependent variable, group as the categorical variable and the RCPM score as the covariate. These analyses were preceded by an overall ANCOVA with the score on the experimental task as the dependent variable, Group as a between-subjects factor, Task (control or emotional) as a within-subjects factor, and the RCPM score as a covariate. There was a Group effect ((*F*(1,89) = 8.798, *p* = 0.004, *η_p_^2^* = 0.089)) but this main effect was qualified by a significant Group x RCPM score interaction ((*F*(1,89) = 8.784, *p* = 0.004, *η_p_^2^* = 0.089)). There was also a significant effect of Task ((*F*(1,89) = 6.440, *p* = 0.01, *η_p_^2^* = 0.067)) but no significant interaction effect involving Task. Finally, the three-way interaction between Group, Task and the RCPM score was not significant (*F*(1,89) = 0.493, *p* = 0.48, *η_p_^2^* = 0.005). Therefore, the Group x RCPM score interaction effect does not vary across the task type. 

#### 3.2.1. Trajectories for the Emotional Task

**Figure 3:**
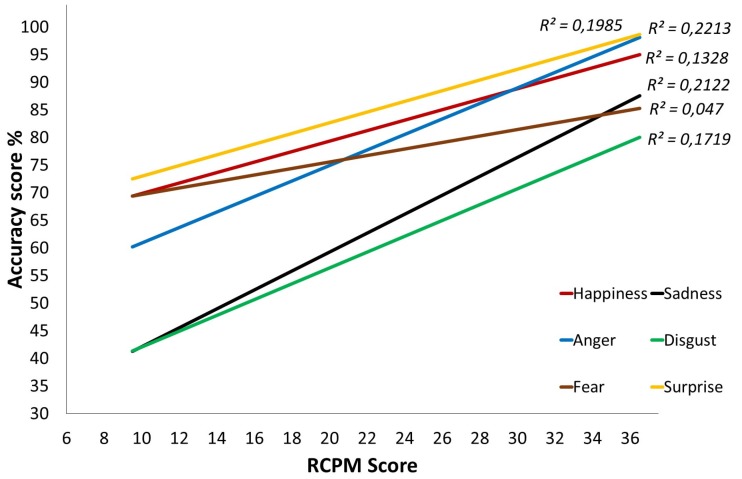
Cross-sectional developmental trajectories for the six basic emotions over Raven’s Colored Progressive Matrices (RCPM) scores, Typically Developing (TD) group.

**Figure 4:**
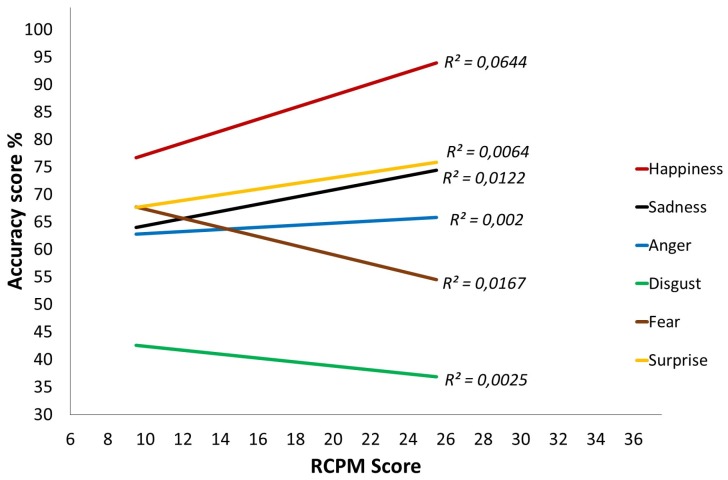
Cross-sectional developmental trajectories for the six basic emotions over Raven’s Colored Progressive Matrices (RCPM) scores, Down syndrome (DS) group.

Examination of the results on the emotional task indicates that there is no Group effect (*F*(1,89) = 2.441, *p* = 0.122, *η_p_^2^* = 0.027), but there is an interaction between Group and the RCPM score (*F*(1,89) = 4.014, *p* = 0.048, *η_p_^2^* = 0.043). This interaction indicates that the RCPM score predicts success on the emotional task significantly less accurately in the DS group than in the TD group. The same pattern emerges when one studies the recognition of each emotion separately. For the TD group (Figure 3), emotion recognition progresses significantly as the RCPM score increases (*ps* < 0.002), except in the case of fear, for which the significance threshold is not reached (*p* = 0.073). As for the DS group (Figure 4), at the onset, few differences from the TD group are found except in the case of sadness. However, the gradients are clearly flatter and even sometimes reversed, which indicates a progression that has only a weak relationship with the RCPM score. This is confirmed by the linear regression analysis: no gradient differs significantly from 0, which indicates that the RCPM score is not a reliable predictor of successful recognition of the six basic emotions by the DS group.

#### 3.2.2. Trajectories for the Control Task

Within each group, the slopes representing each task (control and emotional) are fairly parallel (Figure 2). Nevertheless, at the lowest RPCM score, the DS group had a clearly higher percentage of successes than the TD group. Moreover, for the DS group, performance on the control task did not change in association with the increase in the RCPM score, whereas there was a major change for the TD group. This is confirmed by the ANCOVA: a significant Group effect confirms this difference at the intercept (*F*(1,89) = 11.005, *p* = 0.001, *η_p_^2^* = 0.110), and the difference in slopes is confirmed by the significant Group x RCPM score interaction (*F*(1,89) = 8.194, *p* = 0.005, *η_p_^2^* = 0.084). In the DS group, the RCPM score does not predict success on the control task, contrary to what is found for the TD group.

## 4. Discussion

The objective of this study was to examine the basic emotion recognition abilities of a group of adolescents with DS in comparison with a group of TD children matched for nonverbal reasoning level. The recognition of emotional facial expressions was studied with a nonverbal task in which participants had to watch a short video clip and associate the prosody of a non-emotional sentence presented auditorily with an actor’s facial expression. To better understand the factors involved in the process of recognizing emotional facial expressions for people with DS, all use of emotional vocabulary was eliminated and the task was designed to avoid overloading working memory.

### 4.1. Discussion of Results Obtained in Comparison of Individually Matched Groups

The overall success level for emotion recognition in adolescents with DS was at the same level as in TD children. Nevertheless, unlike the TD children, the DS group obtained better results on the control task than on the emotional task. When the results for each emotion were analyzed separately, no difference between the two groups appeared: the adolescents with DS recognized fear, surprise and anger as well as their TD counterparts. The lack of difference in this emotion recognition task corroborates Channell et al.’s [23] study conducted with children with DS by means of dynamic stimuli (video clips). It also corroborates the study by Pochon and Declercq [24] of children with DS, which made use of static stimuli (photos). On the other hand, the results differ from those observed in earlier studies that used static stimuli, along with emotional vocabulary [9,10,14,15,25] or with dynamic stimuli and production of emotional vocabulary [27]. The original feature in this study was the design of a so-called “nonverbal” task, in the sense that language comprehension and production, particularly of emotional words, was not mobilized in any way for the recognition of emotions. As well, the emotions were presented in the form of film clips, which remains rare in studies of emotion recognition, especially in the case of DS. Finally, the cognitive load induced by the formulation of responses was controlled—at each presentation, a single distractor was presented simultaneously with the target emotion. This choice of methodological precautions led to results suggesting that adolescents with DS are not affected by a specific emotional knowledge deficit relating to the basic emotions.

As in the study by Pochon and Declercq [24], the non-use of emotional vocabulary in the instructions and the formulation of responses undoubtedly avoided certain errors related to insufficient mastery of emotional vocabulary. In this regard, Williams et al. [18] emphasized that certain problems experienced by some of the participants in their study might have been related to a poorer understanding of language even though their verbal level was monitored. Similarly, Kasari et al. [9] mentioned that children with DS, in conversation with their mothers, may have been less exposed to words relating to internal states and therefore might have a less extensive emotional vocabulary [40]. In addition, the administration of tasks that make use of dynamic stimuli—which are more ecological—appears to be more appropriate for assessing participants with DS than traditional tasks with static stimuli; that is true of this study and the one by Channell et al. [23], which did not find evidence at that time of the deficits mentioned [9,10,17,18,25]. Nevertheless, Martínez-Castilla et al. [27], despite their use of animated faces (morphing), found that participants with DS had more problems than TD participants and those with WS, but as they point out, the task was a labeling task, which requires productive verbal skills. Thus, the participants with DS could have been at a disadvantage.

### 4.2. Discussion of Results for the Study of Cross-Sectional Developmental Trajectories

Regarding developmental trajectories, this study shows an unexpected development profile in adolescents with DS. Our results indicate that in this group, success on the RCPM is not a valid predictor of the nonverbal recognition of emotions, whereas it is a completely satisfactory predictor for TD children. Still, the RCPM [34] was chosen as a preliminary measure of the nonverbal level based on the nonverbal nature of the experimental tasks recommended by Moore [22]. Also, and in addition to its ease of administration, the psychometric and developmental properties of the RCPM mean that it is widely used with both TD participants and those with a disorder [41,42]. Moreover, the lack of association between nonverbal reasoning and success on the experimental task was not specific to the processing of emotional information, since it was also observed for the control task. This finding was unexpected since Facon and Nuchadee [42] found the same pattern of responses to the RCPM items in children and adolescents with DS, compared to children and adolescents with ID of undifferentiated etiology and TD children. Consequently, it would be hard to claim that a specific kind of nonverbal reasoning is involved in the RCPM to explain the lack of correlation with success on nonverbal tasks in adolescents with DS.

It would be preferable to hypothesize that in the tasks used in our experiment, participants with DS applied types of cognitive processing that were unrelated to those they had used to respond to the RCPM items, whereas the TD children did not. Specific studies will be necessary to determine the nature of these processes. For example, adolescents with DS could have used a verbal recoding of the visuo-spatial stimuli while this strategy was not yet available in TD children given their young age. This leads to the more general question of the role of life experience; as the DS participants were older than TD children, perhaps they could process emotional information differently. Furthermore, examination of the literature on emotion recognition in DS reveals highly variable results from one study to another when it comes to the link between matching measures and success on experimental tasks. Although Williams et al. [18] found no link for a match based on a short form of the WPPSI-R [43], Pochon and Declercq [25] found a significant correlation for a match based on the nonverbal K-ABC [44], as did Channell et al. [23] for one based on the PPVT-4 [26]. There again, studies specifically focusing on this question will be able to establish whether or not there is a specific characteristic in DS affecting nonverbal information processing.

### 4.3. General Discussion

This study does not provide sufficient evidence to conclude that adolescents with DS have no basic emotion recognition deficit, but it suggests as much. Our hypothesis that the overall recognition level would be lower in this syndrome was not confirmed, nor was the hypothesis that participants with DS would be poorer at recognizing expressions of fear, surprise and anger. Nevertheless, one result raises questions: participants with DS performed worse on the emotional task than on the control task, which was not the case for TD participants. Both experimental tasks were rigorously constructed and presented in the same way and they differed only in the nature of the stimuli used: the auditory and visual stimuli were nonhuman and non-emotional in the control task, contrary to the contents of the emotional task. One might therefore wonder whether the emotional nature of the stimuli makes the task more complicated for adolescents with DS than for TD children, which would explain the greater decline in their scores from one task to the other. This finding raises questions about their abilities to process emotional information and therefore constitutes a limitation on our results. Nevertheless, this study constitutes a major methodological advance, which shows that it is possible to effectively evaluate emotional facial expression recognition without using emotional vocabulary, which is essential in DS given the substantial language problems associated with this syndrome [31]. As for the lack of correlation between RCPM scores and success on the experimental tasks, it constitutes both an essential piece of information about the cognitive and developmental characteristics of people with DS and a limitation on the strength of our results. Since Raven’s Progressive Matrices were used as the basis for matching in our comparative study, it would have been desirable to find a significant correlation between this measure and the experimental results in both groups. It would therefore be relevant to replicate this study using a different matching measure such as receptive vocabulary level since, according to some authors [45,46], vocabulary level tends to correspond to nonverbal cognitive level in children with DS. The PPVT-4 [26] was used effectively by Channell et al. [23] in their study of emotional knowledge. Finally, we must point out that we did not have reference data for our emotion recognition task as no study of standardization has been done in DS, which limits the generalization of our results.

## 5. Conclusions

The findings of this study, which focused on emotion recognition, should enhance our knowledge of emotional competencies in children and adolescents with DS. It makes a significant contribution to a better understanding of the emotional development of people with DS, in an age group that has been not been studied exhaustively, even though major developmental changes take place during this time [47]. Important direction for future research is to examine developmental changes across the life span. Our study could thus be extended to adults with DS. It should be complemented by studies of emotion comprehension [48] and of the link between Theory of Mind, emotional abilities and social understanding [49]. Currently, there is a serious lack of information in these fields, so increasing our knowledge of the development of emotional competencies is essential in order to better target educational and psychological interventions for people with DS.

## Figures and Tables

**Table 1:** Characteristics of Raven’s Colored Progressive Matrices (RCPM) Matched Groups.

Variables	Group				*t* value
	Down Syndrome		Typically Developing		
	**Mean**	**SD**	**Mean**	**SD**	
Chronological age	176.70	28.57	53.14	7.75	20,44 ***
RCPM raw score	15.25	5.17	15.29	4.59	−0.03
BFRT score	31.00	3.35	31.78	3.07	−0.83
Cubes (WPPSI-III) age equivalent	53.91	14.63	53.14 ^†^	7.75 ^†^	-

Note. *n* = 24 in each group. Ages are reported in months. * *p* < 0.05. ** *p* < 0.01. *** *p* < 0.001; ^†^ Typically developing (TD) children were not given this test, chronological age is reminded for comparison purpose. BFRT = Benton Facial Recognition Test; WPPSI = Wechsler Preschool and Primary Scale of Intelligence.

**Table 2:** Number of appearance of the key elements contained in the video stimuli.

Control Task		Target Videos	Distractor Videos	Emotional Task		Target Videos	Distractor Videos
Player	Metal spoon	9	9	Actor	Woman	9	9
	Wooden spoon	9	9		Man	9	9
Sentence	Three blows	6	6	Sentence	Léa est venue en avion *	6	6
	Two double-blows	6	6		La bouteille est sur la table **	6	6
	Two triple-blows	6	6		Cognogo tiketou ***	6	6
Object	Spatula	3	3	Emotion	Happiness	3	3
	Juicer	3	2		Sadness	3	3
	Bottle	3	4		Anger	3	3
	Cooking pot	3	3		Disgust	3	3
	Glass	3	3		Fear	3	3
	Bowl	3	3		Surprise	3	3

* Léa came by plane; ** The bottle is on the table; *** Without meaning.

**Table 3:** Emotional scores in Raven’s Colored Progressive Matrices (RCPM) matched groups.

Emotions	Max. score	Group			
		Down Syndrome		Typically Developing	
		**Mean**	**SD**	**Mean**	**SD**
Happiness	3	2.50	0.66	2.17	0.76
Sadness	3	2.04	0.91	1.46	1.06
Anger	3	1.92	0.65	1.96	0.99
Disgust	3	1.21	1.10	1.42	0.83
Fear	3	1.88	0.99	2.21	0.78
Surprise	3	2.13	0.99	2.33	0.64
